# In-Field Habitat Management to Optimize Pest Control of Novel Soil Communities in Agroecosystems

**DOI:** 10.3390/insects8030082

**Published:** 2017-08-05

**Authors:** Kirsten A. Pearsons, John F. Tooker

**Affiliations:** Department of Entomology, The Pennsylvania State University, University Park, PA 16802, USA; tooker@psu.edu

**Keywords:** tillage, cover crop, soil community, pest control, in-field habitat, novel ecosystem, soil insecticides, seed treatments

## Abstract

The challenge of managing agroecosystems on a landscape scale and the novel structure of soil communities in agroecosystems both provide reason to focus on in-field management practices, including cover crop adoption, reduced tillage, and judicial pesticide use, to promote soil community diversity. Belowground and epigeal arthropods, especially exotic generalist predators, play a significant role in controlling insect pests, weeds, and pathogens in agroecosystems. However, the preventative pest management tactics that dominate field-crop production in the United States do not promote biological control. In this review, we argue that by reducing disturbance, mitigating the effects of necessary field activities, and controlling pests within an Integrated Pest Management framework, farmers can facilitate the diversity and activity of native and exotic arthropod predators.

## 1. Introduction

In natural or semi-natural systems, habitat management focuses on conservation and restoration to strike a balance between resources for wildlife and humans [1]. In agroecosystems, habitat management focuses on providing food and shelter for agronomically beneficial species, especially pollinators and natural enemies, without compromising crop yield and quality [2]. One of the challenges of habitat management in agroecosystems is that there is no baseline, sustainable state to which we are trying to “restore” agroecosystems—they exist to provide human resources, so it is hard to set a balance point between wildlife and human use.

To further complicate habitat management in and around crop fields, agricultural landscapes are dominated by novel habitats because the foundation of agroecosystems, the crops themselves, are almost always exotic: nearly 90% of crops grown in the United States originated from elsewhere in the world [3]. As a result, crop fields harbor mixed arthropod communities, comprising species that share evolutionary histories with either the crop species or with adjacent or nearby habitats, and many species that are entirely alien to the crop and the landscape; these exotic species are either adapted to frequently disturbed systems or particular stages of succession [4]. Because of this range of evolutionary histories, arthropod communities in agroecosystems are challenging to manage sustainably, particularly when yield and crop quality are the ultimate goals; however, by considering the evolutionary histories of species common to crop fields, we can gain insight on approaches to managing agricultural habitats to optimize the function of novel mixed-history communities that they support. Given that exotic arthropods tend to be more prevalent within crop fields than in adjacent habitats, in-field practices should hold greater value for promoting arthropod-associated functions.

This review will focus on the influence of in-field crop and habitat management practices on the novel epigeal and subterranean arthropod communities typically found in row-crop fields (but our arguments should also generally apply other types of agriculture); these arthropod communities play significant roles regulating agroecosystem functions [5]. Extensive work has shown how diverse and abundant soil communities are necessary to optimize nutrient cycling, carbon sequestration, and soil-water dynamics in agroecosystems [5,6,7,8,9]. We will focus on the less-acknowledged role of soil communities in pest suppression and touch upon habitat management practices that help build these communities while being compatible with other ecosystem management goals—especially practices which promote soil quality.

Soil-borne pests, including insects, are often difficult to monitor and control because of the logistics of sampling their populations or deploying control tactics [10]. Therefore, biological control is especially valuable for keeping these often-inconspicuous pest populations in check and below economic thresholds [11]. Unfortunately, popular in-field tactics for controlling soil pests, including tillage or soil-applied insecticides, can be highly disruptive and negatively influence the functional diversity of soil communities [12,13,14,15,16]. A loss of functional diversity in turn reduces the value of soil communities as allies against pests and as regulators of biogeochemical processes [17]. It is especially concerning that popular control tactics, like insecticide use, often go unrecognized as disruptive and are usually employed without knowledge of pest pressure—hindering the soil community with minimal pest control benefits [18,19,20]. Long-term, successful management of soil pests will rely on proven conservation tactics, like avoiding tillage and planting cover crops, in conjunction with a return to Integrated Pest Management (IPM), to maximize benefits provided by the novel soil communities that populate crop fields.

## 2. A Focus on In-Field Practices to Promote Soil Community Diversity in Agroecosystems

In-field management has advantages over landscape-level management due to the novel nature of soil communities in agroecosystems and the challenges that come with implementing landscape-level management.

### 2.1. In-Field Soil Communities are Novel

The invertebrate communities of agricultural soils, especially in annual cropping systems, are novel; they partially comprise local species, but a significant portion of these communities are exotic [4]. Some of these exotic species share an evolutionary history with crop species, while many are entirely alien to both the native landscape and crop species. For example, the community associated with maize cultivation in Pennsylvania and much of the eastern Corn Belt of the U.S. includes many native species and many species, both pests and beneficial, that are exotic to both the landscape and the crop (Table 1).

The complexities of these highly-mixed communities are often overlooked, and habitat management strategies tend to focus on protecting and establishing semi-natural habitats such as hedgerows and pollinator strips [21]. Such off-field manipulations are thought to bolster the in-field activity of local species, improving arthropod-driven functions such as pollination, pest suppression, and nutrient cycling. Off-field management can often improve in-field biodiversity and may increase crop yields [22]; however, in some cases off-field habitat manipulations fail to improve pest management [23]. Native species alone, some of which are adapted to local grassland or forest ecosystems, may not be the best regulators of agroecosystem function. For example, natives can be poor colonizers of agricultural fields and may never establish significant in-field populations [24]. As noted above, the predator, pollinator, and decomposer communities in agricultural fields deviate significantly from those found in adjacent natural areas, and comprise a higher proportion of exotic species [25,26,27]. While these exotic, invasive species may displace native species, their net effects on ecosystem services in agricultural systems may still be positive because they may be more suitable to function under the novel conditions of agroecosystems [28]. Moreover, beyond the direct benefits provided by exotics in agroecosystems, some native species that can establish in-field populations will benefit from interactions with co-occurring exotic species [29,30]. By recognizing that arthropod communities in agricultural soils are novel assemblages that differ from those in local natural soil communities [31], researchers and pest managers can identify in-field tactics that facilitate beneficial interactions between native species and highly cosmopolitan exotics to better optimize ecosystem services in agroecosystems. Because agronomic soils account for 40% of land area, it is unsustainable to assume the remaining 60% of land can overprovide necessary biogeochemical services [32]; thus, it is necessary to optimize the services provided by the novel soil communities in agronomic soils.

### 2.2. In-Field Practices are More Feasible

The majority of agricultural landscapes in the United States are homogeneous: they are dominated by a small number of intensively managed crop species. In 2016, over two-thirds of crop acres in the country were planted with just three crop species, corn, soybean, and wheat [33]. Because heterogeneous agricultural landscapes support increased ecosystem functioning, including predation [34], homogeneous agricultural landscapes can be viewed as somewhat deficient. An ideal heterogeneous, agricultural landscape is patchy, with semi-natural, natural, and agricultural habitats interspersed across the landscape [35]; however, efforts to make homogeneous landscapes more heterogeneous would obviously be challenging and may be unlikely to occur. Such efforts would require high levels of cooperation and intervention among farmers, governmental agencies, and municipalities, but these efforts may be beyond the reach of typical farmers and are better addressed with collaboration among stakeholders. Even if farmers take off-field initiatives to increase local spatial diversity by adding features like hedgerows or pollinator plantings [36], these efforts may have little effect because of the overwhelming homogeneity of the greater landscape [23]. Moreover, the value of these efforts may diminish if other control practices, such as widespread use of prophylactic pesticides, restrict their benefits [23,37,38].

Farmers can, however, more easily implement efforts directly in their fields. In-field practices can help compensate for the lack of off-field habitat in homogenous landscapes [34,39] and it gives farmers the power to facilitate ecosystem function from which they will directly benefit in the long term—reduced inputs, increased yield, and increased profit [40,41,42]—without having to take land out of production [2].

## 3. Roles of Arthropods in Pest Management

Although popular management practices limit the potential for insects, spiders, and other arthropods to control pests [19,43,44], arthropod predators can still provide economically significant pest control services, especially with well-executed habitat manipulations. However, putting a total dollar value on their management of invertebrates, weeds, and pathogens has proved challenging and varies significantly across crop type and agronomic practices [45].

### 3.1. Controlling Invertebrate Pests

It has long been recognized that insects and their kin are powerful regulators of invertebrate pest populations [46]. Research has revealed examples of predator species controlling specific insect pest populations, both aboveground and belowground [11,47], and much is known on the value of generalist arthropod predators for suppressing multiple pest populations below damaging levels [28]. Insect predators are estimated to contribute $4.5 billion of insect pest control services in the United States, a greater annual value than the entire corn harvest of Indiana (the country’s fourth state in corn production; [21,48]). Because this estimate excludes non-insect predators (e.g., spiders, harvestmen, centipedes) and non-insect pests (e.g., slugs, mites), the value of generalist arthropod predators is likely even greater. This invaluable contribution from arthropod predators would only increase if adoption of reduce tillage, cover crops, and judicial pesticide use becomes more widespread.

### 3.2. Controlling Weeds

Weed seed predators, especially carabid beetles, can help reduce weed pressure, although their influence on the weed seed bank is often subtle in the short-term [44,49]. Unlike with insect pests, there are no reliable estimates of invertebrate contribution to weed control [21]. Regardless, management practices that concentrate weed seeds on the soil surface (such as no-till) can facilitate higher weed-seed predation rates [50].

### 3.3. Controlling Pathogens

While most insect-plant-pathogen interactions involve vector-borne diseases, there are multiple ways arthropods can reduce pathogen risk to plants. Most obvious is natural-enemy-driven suppression of vector populations, which reduce the likelihood of pathogen transmission [51]. Soil arthropods can also promote disease suppressive soils by enhancing belowground microbial diversity [9]. Additionally, there is some evidence that diverse and abundant saprophytic arthropods, such as collembolans and mites, will directly feed on fungal root pathogens and reduce plant infection rates [52].

### 3.4. Benefist of Exotic Generalist Predators

Exotic generalists commonly dominate the predator communities of agroecosystems. Although exotic generalist predators may locally displace resident generalist predators, their net effect on biocontrol of herbivores is often still positive. Exotic generalist predators are often more capable of thriving in disturbed sites and they may be uniquely equipped to control non-native pests that native predators cannot handle very well [28]. Also as generalists, some exotic species can contribute broader community functions than specialist predators, so communities containing exotic generalist predators may maintain high functional diversity despite relatively low predator diversity.

## 4. Tactics for Controlling Belowground Pests and the Value of IPM

There are two general strategies for choosing managing tactics for controlling pests. The first, and currently the dominant strategy being used in field-crop production across the U.S., is preventative and relies on deploying pest management tactics regardless of need [53]. The second strategy is holistic, ecologically based, and relies on IPM to deploy management tactics when appropriate.

Regardless of the strategy that farmers use, there is a limited number of control tactics available for belowground pests, which tend to be challenging to control. The short list of control tactics includes tillage, crop rotation, transgenic insect-resistant crop varieties, and soil-applied or seed-applied insecticide. Tillage has been used for centuries to control pests, including weeds, insects, and other invertebrates, such as slugs, but regularly disrupting soil decreases its quality and leaves it susceptible to erosion [54,55,56]. Crop rotation is a stalwart of IPM, and remains a reliable approach for disrupting pest lifecycles [56,57,58,59]. Moreover, there is strong evidence that more diverse rotations have fewer pest populations [58,60]. Soil-applied insecticides have been available for decades and have been used to manage some important belowground corn pests, like *Diabrotica virgifera virgifera* (western corn rootworm). Unfortunately, over-reliance on soil insecticides for controlling *D. virgifera* has hastened evolution of resistance to these insecticides by this key pest species [61]. Transgenic insect-resistant crop varieties (i.e., *Bt* corn) targeting rootworms were introduced in part to overcome challenges associated with insecticide resistance [61,62], but deployment of this technology has been less than perfect and populations of *D. virgifera* have evolved resistance in many parts of the Corn Belt to at least two of the traits deployed against it [63,64,65]. Lastly, neonicotinoid seed coatings are relatively new tools that have been rapidly adopted in field-crop production [53]. These seed treatments mostly target secondary pest species whose populations are usually below economic thresholds and are difficult to monitor or predict. While neonicotinoid seed coatings may have more value in regions of the U.S. with more predictable secondary pest pressures [66], mounting evidence suggests that seed coatings can have significant environmental and non-target effects that may be overshadowing their benefits. Neonicotinoid seed treatments have been implicated in polluting water, increasing mortality of pollinators, reducing natural enemy populations, and even exacerbating some invertebrate pest populations [19,67,68,69,70]. With these significant environmental impacts, it is especially concerning that seed treatments tend to provide limited and inconsistent yield benefits because the pests they target are often uncommon [20,58] and the overuse of a limited set of active ingredient as seed treatments will only accelerate pesticide resistance [71].

Many corn farmers in the U.S. use a preventative strategy—often without knowledge of pest pressures—to manage belowground insect pests, typically using tillage, transgenic insect-resistant seeds coated with neonicotinoid insecticides, and some of this acreage, particularly where continuous corn is grown, will also see a soil-applied insecticide to help control *D. virgifera*. For soybean production, which has less severe belowground pests, farmers will use tillage and insecticidal seed coatings. In portions of the country where no-till agricultural is common for erosion control and nutrient management, like in Mid-Atlantic states and along major rivers of the Midwest, tillage of course would not be used.

In our view, these no-till fields are an important form of in-field habitat manipulation that can serve as the base for an alternative strategy to belowground pest management, which is holistic, ecologically based, and relies on IPM. Although there is a need for more basic ecological research on soil pests to understand why they are problematic under various conditions [59,72], first there is a need to increase implementation of management tactics for soil pests that are already proven and compatible with biological control [58]. Within an IPM framework, insecticide use is governed by an understanding of local pest populations and economic thresholds for pest species [73]. Such deliberate use of insecticides will maintain populations of beneficial species that can be allies in pest control and reduce the risk of pests evolving resistance to insecticides [58,74].

## 5. In-Field Management Strategies to Optimize Arthropod Predators’ Control of Pests

While not all arthropod predators will benefit from the same conditions, agroecologists recognize that certain practices can promote arthropod predator activity [47]. Due to their trophic position, predators are particularly sensitive to agricultural practices, especially cultivation and insecticide use [75]. In certain cases, mechanical and chemical control tactics can be so hard on arthropod predators that overall pest pressures actually increase [19,58]. There are, however, two main types of practices that can promote populations of arthropod predators: practices that reduce management intensity—e.g., reducing tillage—and practices that help compensate for necessary management—e.g., cover crop adoption. But benefits to be gained from the habitat manipulations of no-till and cover crops can best be achieved and maximized if farmers use Integrated Pest Management to decrease unnecessary, and potentially disruptive, insecticide use [58,76].

### 5.1. Balancing Disturbance and Stability to Optimize Biocontrol

In any ecological system, it is impossible to completely eliminate disturbance, even more so for agroecosystems. On top of the abiotic disturbances that natural systems face, annual cropping systems also face a range of field practices (e.g., planting, fertilizer application, pest control activities, harvest) that disrupt the soil community. This more intense and frequent disturbance regime means communities that thrive in agroecosystems can often tolerate more disturbances than the adjacent semi-natural community. This is why pest complexes in annual cropping systems tend to be dominated by exotic, invasive pests that have an evolutionary history with the crop species or are able to tolerate higher levels of disturbance. This latter point also means that the predators that will have the chance to most effectively control these pest species are likely to be disturbance-tolerant invaders themselves [77].

High frequency of disturbances also means that farmers need to rely on management practices that promote diversity and stability to promote a community that functions more like those in non-agricultural habitats. In highly disturbed systems, most predators have to move in from surrounding areas because even disturbance-tolerant species often cannot survive the full management cycle in crop fields [77]. Additionally, natural-enemy populations are likely to be low in extremely simplified landscapes because there are few natural areas that can be sources of mobile predators. However, overwintering within the field can be facilitated with no-till, cover crops, reduced pesticide exposure, and increasing resource diversity, all of which may combine to maintain disturbance-tolerant, non-native predators without having to rely on the complexity of the surrounding landscape [34].

### 5.2. Reducing Disturbance

While some level of disturbance in annual cropping systems is inevitable (e.g., planting, harvest), reducing the frequency and intensity of management activity will promote greater density and diversity of beneficial invertebrates [56,78]. No-till fields provide stable habitats well known for their capacity to build and harbor populations of earthworms and other beneficial invertebrates, and overall pest pressure is most likely to stay constant or even relax in reduced till systems [56]. This absence of growing pest populations appears to be modulated by increased density and diversity of generalist predators in no-till systems, which provide more stable habitat and food sources throughout the year. However, unnecessary insecticide use can reduce the density and diversity of generalist predators in no-till crop fields, counteracting the benefits of no-till adoption and giving the appearance of greater pest pressures in no-till systems [79]. So adopting one predator-promoting practice, such as no-till, may lose its value if another management tactic or tool, (e.g., insecticide use) counteract any potential benefits [58].

### 5.3. Compensating for Unavoidable Disturbance

Other habitat management strategies can be used to compensate for necessary field activities. A critical compensatory practice is ensuring that fields experience diverse plant cover for most of the year through use of cover crops, mulching, intercropping, and tolerance of low weed pressure [47,76,80]. Ground cover—whether in the form of cover crops, weeds, or crop residues—provides continuous shelter and resources for natural enemies [81]. Instead of having to rely on movement of natural enemies from floral strips or hedgerows, systems with diverse plant cover provide on-site sources that natural enemies need to thrive and control pests. Greater in-field crop diversity, including the use of cover crops, crop rotations and permitting low weed pressure, will increase arthropod biodiversity, notably specific beneficial groups such as carabid beetles [28,82].

Unfortunately, the shelter and resources provided by cover crops can also benefit certain pests (e.g., slugs and armyworm, *Pseudaletia unipuncta* [Haworth]), which may become more of a concern when cover crops are adopted in concert with reducing tillage [54,83]. However, as discussed in the next section, farmers using IPM can deal with these pest populations indirectly by diversifying rotations or changing cover-crop species or directly via scouting and using rescue treatments where necessary [56].

### 5.4. Compatability with the Goals of Integrated Pest Management

Most critically, pest-control tactics should be compatible with the goals of IPM. Such tactics should enhance, or at least not disrupt, biological control, and they should work in concert, not against each other [73]. For example, cover crops can help compensate for yield losses due to higher weed pressure in reduced tillage organic systems [42], and can foster even more natural-enemy activity, leading to greater predation of key pest species [76,80,84]. However, the functionality of no-till and cover crops largely relies on maintaining robust soil communities, which prophylactic insecticide use may retard. Importantly, the IPM framework creates a management environment in which farmers can build stable in-field habitats that will facilitate a mixed community of native and exotic beneficial arthropods ready to attack growing pest populations. Moreover, IPM can be adapted to different systems with unique pest pressures and management requirements. Adopting cover crops and eliminating tillage will not work for every crop, climate, and geological landscape [85,86], but farmers can work within an IPM framework to adopt other in-field tactics that reduce disturbance and mitigate for field activities. Most importantly, IPM can positively affect the whole ecosystem beyond pest management by avoiding unnecessary reductions in pollinator and decomposer activity [87,88,89,90,91,92] or pollution of local waterways [68,93,94,95].

## 6. Value of Relying on Multi-Purpose Management Practices

While pest management strategies that rely on chemical inputs are rather one-dimensional, holistic habitat management provides benefits beyond pest control. Off-field manipulations such as hedgerows and flowering borders provide “stacked” ecosystem services—pollinator habitat, natural-enemy habitat, erosion control, and aesthetic value [96]. In-field practices, like reduced tillage and use of cover crops, also provide stacked services, and are traditionally first adopted for erosion control, improving soil quality, and nutrient management before their pest control benefits are even considered [80,81,97,98,99]. Even if in-field practices are adopted for just one of these benefits, the other benefits remain and often stem from increased community diversity [100]. Reduced tillage and cover crops have even been identified as valuable tools for mitigating and adapting to elevated carbon dioxide and climate change [101], further evidence that these practices are more likely to have positive externalities, unlike the environmental trade-offs typical of prophylactic pesticide use and tillage. One of the key “other” services provided by the soil community is decomposition, which is vital for residue breakdown and nutrient cycling. Conserving natural enemies with no-till and cover crops as a part of IPM also protects the invertebrate species responsible for decomposition, further linking pest management with nutrient cycling and soil quality [102].

### Decomposers as Alternative Resources for Arthropod Biocontrol Agents

Habitat management practices that directly reduce predator mortality (cover crops, no-till, reduced pesticide use, and residue retention) also facilitate a diverse and robust decomposer community. [87,88,103,104,105,106,107,108]. The decomposer and microbivore community comprises a diversity of mesofauna (e.g., collembola, mites, and encheitraeids) and macrofauna (e.g., earthworms, isopods, and diplopods) which are valuable in their own right since they accelerate residue breakdown and mobilize nutrients [109]. An active decomposer community is even more valuable in no-till systems, where invertebrate activities are necessary for incorporating material and releasing nutrients into the soil [25,104]. More relevant to this review, decomposers provide a valuable alternative food source for generalist predators when pest populations are low [2,110,111,112,113] and when pest populations increase, systems with robust decomposer communities are more resilient to pest outbreaks [114]. Decomposers are especially valuable prey for exotic generalist predators, which are often better than native predators at using alternative prey in times of low herbivore densities [28]. That said, both native and invasive predators benefit from an abundance of alternative prey which can be consumed directly and can reduce the frequency of intraguild predation [30,115].

## 7. Future Research Needs

While there is good understanding that habitat manipulation can foster robust predator populations that can assist with pest control, research gaps remain and these need to be addressed to have a better understanding of how in-field management practices influence communities of beneficial arthropods. With future research, there should be increased focus on how co-adopted management practices affect biotic interactions within the soil community, and how changes to the soil community might affect multiple functions (e.g., pest control and nutrient cycling).

Experiments should be developed to capture the changes to soil communities and ecosystem services when farmers shift from conventional management to reduced tillage, cover crops, and IPM; similar studies have been conducted during the transition from conventional to organic production and can help explain challenges such as temporary yield drag [116]. These future studies should include community-level endpoints (e.g., decomposition, pollination, and pest suppression) as opposed to relying just on measures of relative species abundance because functional diversity and raw biodiversity may not be well correlated in novel agroecosystems with many exotic species [28]. There is also value in investigating the relative contribution of native fauna versus exotic fauna to ecosystem services in agroecosystems—perhaps leading to more recognition and facilitation of beneficial exotics which are already well established across agroecosystems [28,117,118,119,120]. Additionally, we know very little about how prophylactic pesticides interact with cover crops, reduced tillage, and soil quality; it would be valuable to know if beneficial management practices can ameliorate negative effects of prophylactic pesticides, or if these practices are incompatible.

## 8. Conclusions

Holistic management practices, including cover cropping, reducing tillage, and reducing pesticide use, are in-field habitat manipulations that can promote a diverse soil community and should increase arthropod control of pests, reducing reliance on cultivation and pesticide applications. Because the assemblages of arthropods in agricultural fields tend to be novel and do not appear to be found elsewhere, in-field practices are particularly valuable for maintaining exotic generalist predators that significantly contribute to pest control. To maintain predators in fields, there is a need to reduce disturbances caused by field activities, and to compensate for unavoidable disturbances to stabilize the soil community. Belowground pests in particular are challenging to monitor and treat, so control tactics are limited. Management practices that provide balance between stability and disturbance, and are co-adopted with IPM, will optimize the value of biological control services provided by the soil community for control of belowground pests. Most of these management practices, especially reduced tillage and cover crops, provide additional agronomic and environmental benefits that can further increase the resilience of a system to nutrient limitations, pest pressures, and even climate change. Researchers need to explore effects of management activities on the whole soil community and multiple ecosystem services, including the value of a balanced decomposer community for pest management.

To improve the long-term sustainability of farming, there is a strong need for farmers to adopt holistic in-field manipulations that promote functional diversity of soil communities. These communities regulate crucial ecosystem functions, which directly benefit farmers managing their fields. Evidence suggests pesticides are overused and use could be reduced by over 40% without yield or profit loss [121]; adoption of IPM and compatible habitat management practices could further reduce pesticide use. The current challenge is to get more farmers to use IPM. Importantly, this involves working with farmers and their advisors so that they understand how to evaluate pest populations in their fields as well as the strengths and weaknesses of practices that are available to control the invertebrate pest populations that they encounter [122,123], rather than relying on often-unnecessary preventative pest control tactics.

## Figures and Tables

**Table 1:** Common invertebrates found in maize fields in Pennsylvania and the northeastern U.S.

Type of Invertebrate	Native to PA	Exotic Species
Ground Beetles	*Harpalus pensylvanicus*, *Chalaenius tricolor*	*Pterostichus melanarius*
Spiders	*Hogna spp*.	-
Bees	*Bombus spp., Halictus spp., Megachile spp.*	*Apis mellifera*
Slugs	*Deroceras leave*	*Deroceras reticulatum*
Other Pests	*Agroites mancus, Cyclocephala spp., Blissus leucopterus*	*Delia platura, Ostrinia nubilalis*
Other	-	Earthworms, millipedes
